# Ethyl acetate fruit fraction of tetrapleura tetraptera modulates cholinergic anti‐inflammatory pathways: an invivo and insilico study

**DOI:** 10.1002/alz.089895

**Published:** 2025-01-09

**Authors:** Oyinoyi Bethel Onimisi, Joy Ochai, Boniface Salifu, Ubong Udeme Ekpo, Muhammad Sadisu, Sherifat Banke Idris, Gbenga Peter Oderinde, Jibrin Danladi

**Affiliations:** ^1^ Usmanu Danfodiyo University, Sokoto, Sokoto Nigeria; ^2^ Federal University of Health Sciences Otukpo, Otukpo, Benue Nigeria; ^3^ Ahmadu Bello University Zaria, Zaria, Kaduna Nigeria

## Abstract

**Background:**

Studies suggest a potential link between stroke and Alzheimer’s disease wherein stroke may serve as a trigger for the onset or acceleration of Alzheimer’s pathogenesis as damage to the brain’s blood vessels may lead to the accumulation of amyloid beta protein which is a hallmark of Alzheimer’s disease. Recent research has shown that stroke treatment may hold the key to treating Alzheimer’s disease. The anti‐inflammatory potentials of Cholinergic signaling are a novel therapeutic target in memory decline associated with Alzheimer’s. Therefore, cholinergic agonists which play a crucial role in increasing the synaptic levels of acetylcholine could in turn downregulate inflammatory mediators as well as modulate Cholinergic signaling thereby improving cognition

**Method:**

Molecular docking analysis of TT‐Fruits ligand against Iba1(PDB ID: 2d58), IL‐6 (PDB ID: 1Alu), and AChE (PDB ID: 5hfa) was conducted using Pyrex, Autodock Vina option with Galantamine and minocycline (MNC) as a control drugs to evaluate the molecular interactions between the ligands present in EAFFTT and inflammatory mediators implicated in stroke pathology. *In‐vivo* study was conducted as follows: 50 Male adult Wistar rats (200 – 220g) were randomly distributed into five groups: Group I‐Sham, and Group II‐V were subjected to Ischemia/Reperfusion (I/R) for 30 minutes and then treated daily with H_2_O, EAFFTT 200 mg/kg, EAFFTT 100 mg/kg, or MNC 50 mg/kg respectively, orally for seven days. Following this, the brains were extracted and processed for IL‐6 and AChE ELISA and IBA 1 immunohistochemistry for reactive microglia.

**Result:**

molecular docking results predicted Aridanin and Echinocystic acid as the lead ligands in EAFFTT with a higher binding affinity with the proteins (Iba1, IL‐6, and AChE) than the control drugs. In the experimental study, I/R without treatment showed a significant increase in IL‐6, AChE, and the number of activated microglia. However, a significant decrease in IL‐6, AChE, and the number of reactive microglia was observed in I/R +EAFFTT (100 and 200 mg/kg), and I/R +MNC (50 mg/kg) ‐treated groups

**Conclusion:**

*Tetrapleura tetraptera* fruit phenolics docked well with IBA1, IL‐6 and AChE, thereby modulating their expression, as EAFFTT is a potential neurotherapeutic agent against stroke‐induced neuroinflammation

